# Association of healthy eating index-2015 and overactive bladder: a cross-sectional study

**DOI:** 10.3389/fnut.2024.1400398

**Published:** 2024-09-17

**Authors:** Xuanyu Hao, Gang Liu, Dongyang Li

**Affiliations:** ^1^Department of Gastroenterology, Shengjing Hospital of China Medical University, Shenyang, Liaoning, China; ^2^Department of Urology, Shengjing Hospital of China Medical University, Shenyang, Liaoning, China

**Keywords:** HEI-2015, OAB, odds ratio, dose–response, cross-sectional study

## Abstract

**Objective:**

The aim of this cross-sectional study was to investigate the association of HEI-2015 and overactive bladder (OAB) in a large population.

**Methods:**

Data were retrieved from the National Health and Nutrition Examination Survey (NHANES) 2005–2020 datasets. Univariate and multivariate logistic regression were performed to evaluate the association between HEI-2015 and OAB. The restricted cubic spline (RCS) model was conducted to investigate the dose–response relationship.

**Results:**

Totally, this study included 29,206 participants with 6,184 OAB patients among them. The higher continuous HEI-2015 value was independently associated with lower OAB incidence (OR: 0.87; 95%CI: 0.78, 0.98). Similarly, the highest quartile categorical HEI-2015 was significantly associated with a lower OAB odds (OR: 0.72; 95%CI: 0.52, 0.99) when compared with the lowest quartile. The RCS curve also showed a favorable non-linear dose–response relationship between HEI-2015 and OAB.

**Conclusion:**

A higher HEI-2015 had a favorable association with OAB and there was a non-linear dose–response relationship between them. We suggest adherence to the United States diet recommendation as a potential behavioral prevention of OAB. Large-scale long term prospective cohort studies across various regions are needed to verify the findings of this paper.

## Introduction

The term “overactive bladder” (OAB) encompasses a storage symptom characterized by “urgency, with or without urge incontinence, usually with more frequency and nocturia” in the absence of obvious infection or pathology, as defined by the International Continence Society (ICS) in 2002 (1). In the United States, the prevalence of OAB is reported as 17% in men and 30% in women (2). However, OAB is often under-reported in both men and women, with only a fraction seeking treatment (3). It is important to note that OAB can significantly impact the quality of life, disrupt sexuality and even lead to depression or anxiety (4). Initial treatment for OAB typically involves conservative behavioral therapies, such as weight loss, reducing coffee/alcohol intake and performing Kegel exercises (5). However, some cases may not respond completely to anticholinergic medications or botulinum toxin injection, leading to medication-refractory OAB (6). The exact pathological mechanism of OAB is still unclear, although research suggests potential causes such as obesity, aging, oxidative stress, systemic inflammation, high androgen levels, and neurological disorders (7). Notably, while there is some understanding of these factors, few studies have thoroughly explored the relationship between nutrition, dietary indexes, and OAB. Therefore, considering the potential impact on the onset and progression of OAB, diet may play a crucial role among the risk factors.

As reported by Bozkurt et al., a Mediterranean diet, predominantly comprising vegetables, nuts, fish, and fruits with reduced red meat intake, has been suggested to alleviate OAB symptoms (8). A healthy lifestyle invariably integrates a high-quality dietary regimen. Can a healthy diet effectively prevent or ameliorate OAB? While it is conjectured that a Western diet may potentially contribute to OAB. A review in this area reported that the current evidence was not robust enough (9). Observational studies focusing on the connection between individual dietary factors and OAB may be constrained by unobserved confounding, limited causality, and misclassification bias (10, 11). Hence, our aim was to determine the significance of a comprehensive dietary measure in the onset and mitigation of OAB.

The impact of diet on OAB is large. Caffeinated beverages and alcohol can irritate the bladder, while spicy foods and acidic foods may increase bladder sensitivity (9). Dietary fiber may improve bowel function and reduce constipation (9). The Healthy Eating Index-2015 (HEI-2015) serves as a quantitative tool for evaluating dietary quality and aligns with contemporary dietary recommendations from experts at the National Cancer Institute (NCI) and the US Center for Nutrition Policy and Promotion (CNPP) (12). Comprising 13 dietary components, higher scores on the HEI-2015 denote an improved pattern of dietary quality as per the American Society of Nutrition (ASN) guidelines (13). Through the implementation of HEI-2015, we can comprehensively gauge the cumulative impact of several dietary elements. Recent reports suggest that a higher HEI-2015 score may yield benefits in reducing the risk of cardiovascular diseases, Crohn’s disease, and depression (14–16). Furthermore, separate studies have explored the association between HEI-2015 and mortality, yielding encouraging results. A prospective cohort study indicated that HEI-2015 was linked to a lower risk of total and cause-specific mortality (17). While another study highlighted that a high HEI-2015 was associated with post-diagnostic cancer mortality among adult cancer survivors (18). However, evidence remains none regarding the relationship between HEI-2015 and the risk of OAB.

In this context, our study aims to explore the association between HEI-2015 and the risk of OAB in a large cross-sectional population in the United States, employing logistic regression, subgroup analyses, and restricted cubic spline (RCS) curves using national databases. Overall, these analyses may ensure clearer comprehension and emphasize the importance of investigating the potential impact of the HEI-2015 on the risk of OAB within the broader context of dietary habits and health outcomes.

## Methods

### Data source and study population

The data for this study were obtained from the National Health and Nutrition Examination Survey (NHANES, https://wwwn.cdc.gov/nchs/nhanes/Default.aspx#), a nationally representative open-access survey. NHANES collected information on dietary recall, as well as demographic, physical, and medical data in order to assess the nutritional status and health of United States civilians. The survey was initiated and approved by the National Center for Health Statistics (NCHS) ethics review board in the United States, and informed consent was obtained from all participants prior to their interviews.

**Figure 1:**
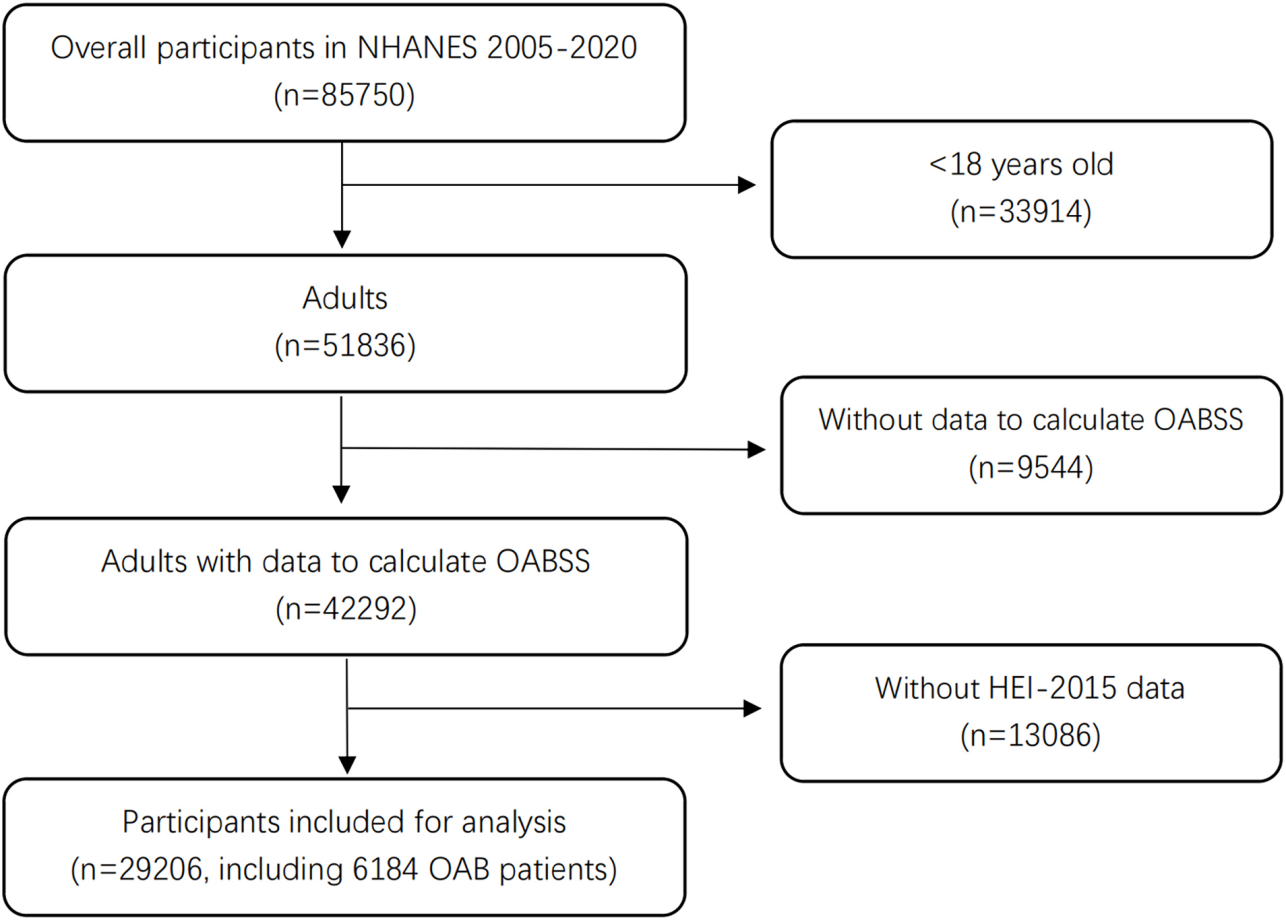
Flow chart of the participant’s collection from the NHANES 2005–2020. NHANES, National Health and Nutrition Examination Survey; OABSS, Overactive Bladder Syndrome Score.

Between 2005 and 2020, a total of 85,750 participants were screened using the NHANES 2005–2020 datasets, covering eight cycles: 2005–2006, 2007–2008, 2009–2010, 2011–2012, 2013–2014, 2015–2016, 2017–2018, and 2019–2020. The inclusion criteria required participants to be over 18 years old and for OAB data calculation as well as HEI-2015 data to be available for calculation. The exclusion criteria included: age < 18 years old, without data to calculate OAB symptom score, without HEI-2015 data. Thus we can select enough participants without other limitations to get objective results. Ultimately, 29,206 eligible individuals were included in the study for further analyses. A flow chart detailing the screening process for participants is presented in Figure 1. This study involved the secondary analysis of publicly available cross-sectional data without personally identifiable information, and therefore, additional Institutional Review Board permission was not necessary.

### Assessment of OAB and HEI-2015

The primary objective of this study was to determine the presence of OAB within the sample population. Diagnosis of OAB was established in accordance with the International Classification of Diseases, Tenth Revision (ICD-10). Exclusion criteria encompassed individuals with benign prostatic hyperplasia, urinary tract infections, or a history of urinary system cancer. Clinical data were obtained through the administration of two questionnaires. Trained evaluators conducted face-to-face interviews with participants, during which they completed the surveys. For instance, the Urge Urinary Incontinence (UUI) score comprised multiple inquiries such as, “During the past 12 months, have you experienced leakage or loss of control of urine due to a sudden urge or pressure to urinate, when you were unable to reach the toilet quickly?” to determine the presence, and “How frequently does this occur?” to assess severity. In the Nocturia score questionnaire, participants were asked, “How often did you get up to urinate in the past 30 days, from the time you went to bed at night to the time you got up in the morning?” to evaluate the severity of nocturia. The Overactive Bladder Syndrome Score (OABSS) was computed by summing the UUI score and Nocturia score. The UUI score value is 0–3 and the Nocturia score value is also 0–3. Therefore, the OABSS value varies from 0 to 6. An OABSS value of ≥3 quantified the existence of OAB.

The main variable of interest was the HEI-2015. According to the Dietary Guidelines for American, the HEI-2015 score was computed from 13 components including whole fruits, total fruits, whole grains, dairy, total protein foods, seafood & plant proteins, greens & beans, total vegetables, fatty acids, refined grains, sodium, added sugars, and saturated fats (13). HEI-2015 scores were derived from total nutrient intakes on the first day (DR1TOT) and the MyPyramid Equivalents Database/Food Patterns Equivalents Database (MPED/FPED) files. However, the HEI-2015 scores were not calculated in intervals. Ranging from 0 to 100, a higher HEI-2015 score indicated greater adherence to optimal dietary recommendations.

### Assessment of covariates

Demographic variables included age (years), ethnicity (Mexican American/Non-Hispanic White/Non-Hispanic Black/other Hispanic/other race, including multi-racial), education (more than high school/high school or equivalent/less than high school), and family income to poverty ratio (PIR), all of which were extracted from self-reported questionnaires.

Behavioral variables encompassed body mass index (BMI—weight in kilograms divided by height in meters squared), smoking status (never/former/current), and alcohol use (never/moderate/heavy). Physical activity (PA) level was classified into low, moderate, and high levels based on the metabolic equivalent of task (MET). Past medical history variables included hypertension (systolic blood pressure > 140 mmHg or diastolic blood pressure > 90 mmHg), diabetes mellitus (DM), and cardiovascular disease (CVD) history. Further details can be found at www.cdc.gov/nchs/nhanes/.

### Statistical analysis

Weighted analyses were conducted in accordance with the NCHS guidelines. The weights in NHANES are generated to account for the complex survey design, survey non-response, and post-stratification to align with total population counts from the United States Census Bureau (19). Each individual in the sample is assigned a specific sample weight that quantifies their representation within the overall population and representative data of the civilian noninstitutionalized resident United States population could be produced by applying the weights (19–21). Initially, baseline clinical characteristics were extracted. Continuous variables with a normal distribution (e.g., age, BMI, and HEI-2015) were reported as means and standard deviations (SD), while the non-normal continuous variables were expressed as median (interquartile range). Categorical variables [e.g., ethnicity, education, PIR, smoking status, alcohol use, hypertension, DM, and CVD] were expressed as cases (*n*) and percentages (%). Statistical comparisons were made using the One-way ANOVA for continuous variables with a normal distribution and the chi-square test for categorical variables across groups, while continuous variables with a non-normal distribution were compared using the Kruskal–Wallis rank sum test.

Subsequently, the association between HEI-2015 and OAB was explored using univariate and multivariate logistic regressions. Effect sizes were expressed as odds ratios (ORs) and 95% confidence intervals (CI). More robust conclusions were drawn through multivariate logistic regression, adjusting for confounding variables in two different models. When the HEI-2015 score was divided into quartiles, the lowest quartile (Q1) was used as the reference. To explore potential biases, subgroup analyses based on BMI, sex, hypertension, diabetes, smoking status, and alcohol use condition were conducted and *p* for interactions were examined. The subgroup analyses were adjusted by the confounders in model 2 of multivariate logistic regression other than variables for stratification. A *p* value for interaction >0.05 in the subgroup analysis indicated that the main result was not impacted by this subgroup. Additionally, a restricted cubic spline (RCS) curve analysis was performed to determine if a dose–response relationship existed between HEI-2015 and OAB. The median value of the HEI-2015 score (54.43) was chosen as the cutoff value. All statistical analyses were performed using R software (Version 4.3.1). A two-sided *p* value less than 0.05 was considered statistically significant.

## Results

### Baseline characteristics of the participants

The behavioral, demographic, and medical history factors of the total 29,206 participants screened from NHANES 2005–2020 are summarized in Table 1. The average age of the total participants was 47.63 years and male participants accounts for 48.17%. Generally, the non-Hispanic black participants (68.02%) and those with over a high school education level (38.54%) were in the majority. Among the entire population, 6,184 participants suffered from OAB. Analysis of the baseline data indicated significant differences in the included variables between different HEI-2015 quartile groups (all *p* < 0.05, except in the CVD group). Participants who adhered to the American nutritional recommendation (HEI-2015 Q4) tended to have higher age (52.09 ± 0.40), lower BMI (27.95 ± 0.12), higher PIR (3.44 ± 0.05), higher education level and were less likely to smoke or drink. The improved dietary quality intake might be associated with a slightly lower risk of diabetes and hypertension, but probably not of CVD only in these study population.

**Table 1:** Baseline characteristics of the whole 29,206 participants from the NAHENS 2005–2020.

Variable	Total	HEI-2015 Q1	HEI-2015 Q2	HEI-2015 Q3	HEI-2015 Q4	*p* value
Age	47.00 (33.00, 60.00)	41.00 (29.00, 55.00)	46.00 (32.00, 59.00)	49.00 (35.00, 62.00)	53.00 (38.00, 65.00)	< 0.0001
BMI	28.00 (24.26, 32.70)	28.90 (24.50, 33.96)	28.42 (24.65, 33.03)	27.80 (24.30, 32.60)	26.93 (23.80, 30.95)	< 0.0001
PIR	3.05 (1.51, 5.00)	2.45 (1.25, 4.29)	2.85 (1.43, 4.95)	3.20 (1.58, 5.00)	3.79 (1.94, 5.00)	< 0.0001
Gender						< 0.0001
Male	14,064 (48.17)	3,935 (54.11)	3,648 (50.99)	3,434 (45.88)	3,047 (41.29)	
Female	15,142 (51.83)	3,367 (45.89)	3,653 (49.01)	3,867 (54.12)	4,255 (58.71)	
Ethnicity						< 0.0001
Mexican American	4,378 (8.39)	935 (8.51)	1,173 (9.22)	1,238 (8.77)	1,032 (7.04)	
Non-Hispanic White	6,292 (11.10)	1,835 (13.57)	1,671 (12.06)	1,530 (10.97)	1,256 (7.65)	
Non-Hispanic Black	13,028 (68.02)	3,520 (68.07)	3,211 (66.68)	3,094 (67.24)	3,203 (70.13)	
Other Hispanic	2,687 (5.19)	505 (4.46)	607 (4.81)	740 (5.50)	835 (6.06)	
Other race	2,821 (7.29)	507 (5.39)	639 (7.23)	699 (7.52)	976 (9.13)	
Edu						< 0.0001
No more than high school	6,844 (21.00)	2,017 (44.88)	1,798 (39.05)	1,640 (32.37)	1,389 (24.66)	
More than high school	9,467 (38.54)	2,093 (55.12)	2,143 (60.95)	2,365 (67.63)	2,866 (75.34)	
Alcohol user						< 0.0001
Never	3,974 (10.73)	811 (11.41)	890 (11.89)	1,055 (13.06)	1,218 (14.71)	
Moderate	9,711 (35.94)	2,082 (36.87)	2,280 (41.11)	2,468 (43.29)	2,881 (49.72)	
Heavy	9,851 (37.40)	2,828 (51.72)	2,710 (47.00)	2,416 (43.65)	1,897 (35.57)	
Smoke						< 0.0001
Never	16,107 (55.58)	3,438 (49.02)	3,812 (53.19)	4,206 (57.62)	4,651 (63.00)	
Former	7,346 (25.00)	1,566 (19.82)	1,803 (24.58)	1,911 (27.16)	2,066 (28.77)	
Current	5,740 (19.40)	2,294 (31.16)	1,683 (22.24)	1,181 (15.22)	582 (8.23)	
PA level						< 0.0001
Low	5,206 (18.00)	1,231 (23.65)	1,330 (25.10)	1,367 (23.77)	1,278 (18.86)	
Moderate	12,885 (48.37)	2,835 (54.49)	3,029 (57.97)	3,196 (62.10)	3,825 (70.08)	
High	3,493 (12.60)	1,183 (21.86)	901 (16.93)	798 (14.13)	611 (11.06)	
CVD						0.14
No	25,886 (91.15)	6,456 (91.38)	6,514 (91.78)	6,447 (90.27)	6,469 (91.18)	
Yes	3,315 (8.84)	845 (8.62)	787 (8.22)	851 (9.73)	832 (8.82)	
DM						0.01
No	23,157 (84.95)	5,948 (87.55)	5,841 (85.68)	5,680 (85.66)	5,688 (85.12)	
Yes	5,506 (13.80)	1,228 (12.45)	1,328 (14.32)	1,481 (14.34)	1,469 (14.88)	
Hypertension						0.02
No	16,521 (61.96)	4,344 (64.51)	4,159 (60.57)	4,080 (61.62)	3,938 (61.03)	
Yes	12,682 (38.04)	2,957 (35.49)	3,142 (39.43)	3,220 (38.38)	3,363 (38.97)	
BMI groups						< 0.0001
< 25	8,011 (29.06)	1,903 (26.90)	1,864 (26.48)	2,018 (29.39)	2,226 (34.51)	
25–29.9	9,582 (32.61)	2,137 (29.19)	2,379 (33.44)	2,463 (33.29)	2,603 (35.64)	
≥ 30	11,352 (37.63)	3,204 (43.91)	2,985 (40.09)	2,765 (37.32)	2,398 (29.85)	
Overactive bladder						0.3
No	23,022 (83.41)	5,807 (84.10)	5,784 (82.69)	5,705 (82.90)	5,726 (83.91)	
Yes	6,184 (16.59)	1,495 (15.90)	1,517 (17.31)	1,596 (17.10)	1,576 (16.09)	

PIR, Family income to poverty ratio; BMI, Body mass index; PA level, Physical activity level; CVD, Cardiovascular disease; DM, Diabetes mellitus. The values are median and interquartile range; number and proportion. The *p* values were calculated from the chi-square test to test the difference among HEI-2015 Q1–Q4.

### Association between HEI-2015 and OAB

The results of univariate logistic regression analyses of each covariate between OAB and non-OAB were demonstrated in Table 2. Based on the non-adjusted model, older/female/lower income/non-Hispanic White/higher BMI/lower physical activity (PA) level participants might probably live with OAB (*p* < 0.0001). Hypertension and diabetes mellitus (DM) were also associated with the risk of OAB (*p* < 0.0001). In addition, smoker or alcohol drinker tended to have a higher risk of OAB (*p* < 0.0001).

**Table 2:** Univariate logistic regression analyses between the covariates and OAB.

Character	OR	95% CI	*p* value
Age	1.04	1.04 (1.04, 1.05)	<0.0001
Gender	1.71	1.71 (1.56, 1.87)	<0.0001
Ethnicity			
Mexican American	ref	ref	ref
Non-Hispanic White	2.07	2.07 (1.79, 2.40)	<0.0001
Non-Hispanic Black	1.11	1.11 (0.97, 1.27)	0.12
Other Hispanic	1.19	1.19 (1.00, 1.42)	0.06
Other race	0.83	0.83 (0.66, 1.03)	0.10
Edu			
No more than high school	ref	ref	ref
More than high school	0.54	0.54 (0.47, 0.63)	<0.0001
Alcohol user			
Never	ref	ref	ref
Moderate	0.67	0.67 (0.57, 0.78)	<0.0001
Heavy	0.52	0.52 (0.45, 0.61)	<0.0001
Smoke			
Never	ref	ref	ref
Former	1.49	1.49 (1.31, 1.70)	<0.0001
Current	1.36	1.36 (1.19, 1.54)	<0.0001
DM			
No	ref	ref	ref
Yes	3.06	3.06 (2.75, 3.40)	<0.0001
CVD			
No	ref	ref	ref
Yes	3.52	3.52 (3.15, 3.93)	<0.0001
Hypertension			
No	ref	ref	ref
Yes	2.81	2.81 (2.57, 3.06)	<0.0001
PIR	0.81	0.81 (0.78, 0.84)	<0.0001
BMI	1.05	1.05 (1.04, 1.06)	<0.0001
PA level			
Low	ref	ref	ref
Moderate	0.79	0.79 (0.68, 0.92)	0.002
High	0.59	0.59 (0.48, 0.72)	<0.0001

PIR, Family income to poverty ratio; BMI, Body mass index; PA level, Physical activity level; CVD, Cardiovascular disease; DM, Diabetes mellitus.

After adjusted by some covariates in two models, the HEI-2015 was regarded as a continuous variable or a categorical variable in the multi-variate logistic regression analyses. Although the HEI-2015 was not significantly associated with OAB in the crude model, a higher HEI-2015 score’s value was associated with the lower risk of OAB in the model adjusted by gender, age and ethnicity (Model 1) and the fully adjusted model (Model 2) (Table 3). When considered as a continuous variable, a higher HEI-2015 could decrease the risk of OAB in the fully adjusted model (Model 3, OR: 0.87; 95%CI: 0.78, 0.98; *p =* 0.02). The participants were subsequently divided into four groups: HEI-2015 Q1 [10, 44.313] with 7,302 (25%) people; HEI-2015 Q2 (44.313, 53.426] with 7,301 people; HEI-2015 Q3 (53.426, 63.066] with 7,301 (25%) people and HEI-2015 Q4 (63.066, 99.5497847692926] with 7,302 (25%) people. Likewise, participants with the highest quartile categorical HEI-2015 was significantly associated with a lower OAB odds (OR: 0.72; 95%CI: 0.52, 0.99) when compared with the lowest quartile.

**Table 3:** Univariate and multivariate logistic regression analyses between the HEI-2015 and OAB.

	Crude model		Model 1		Model 2	
	OR (95%CI)	*p* value	OR (95%CI)	*p* value	OR (95%CI)	*p* value
HEI-2015 continuous	1.01 (0.96, 1.05)	0.83	0.85 (0.81, 0.90)	<0.0001	0.87 (0.78, 0.98)	0.02
HEI-2015 quartile						
Q1	1 (reference)		1 (reference)		1 (reference)	
Q2	1.11 (0.98, 1.25)	0.11	0.94 (0.82, 1.08)	0.39	1.14 (0.82, 1.59)	0.42
Q3	1.09 (0.96, 1.23)	0.17	0.81 (0.71, 0.93)	0.003	0.85 (0.58, 1.26)	0.41
Q4	1.01 (0.88, 1.16)	0.84	0.66 (0.57, 0.76)	<0.0001	0.72 (0.52, 0.99)	0.04
		0.93		<0.0001		0.01

Model 1 was adjusted by “age,” “ethnicity,” and “gender.”

Model 2 was adjusted by “age,” “ethnicity,” “gender,” “edu,” “BMI,” “PIR,” “PA level,” “alcohol user,” “smoke,” “hypertension,” “DM,” and “CVD.”

### Dose–response non-linear relationship analysis

**Figure 2:**
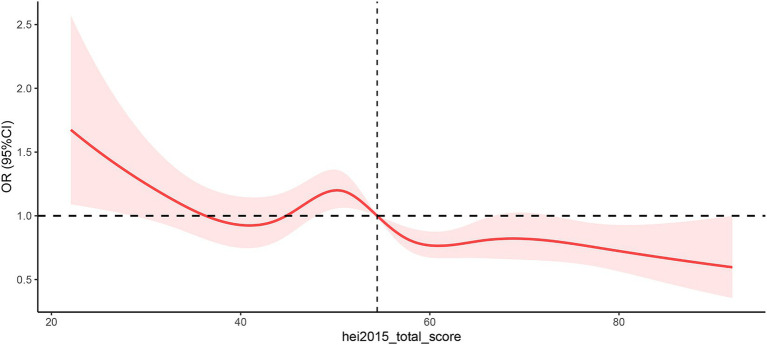
Non-linear restricted cubic spline (RCS) plot between HEI-2015 and OAB. Cut-off value of HEI-2015 = 54.43. OR, Odds ratio. The red line indicates the dose–response relationship between CDAI and BPH and the pink area represents the 95% confidence interval.

The restricted cubic spline method was employed to assess the dose–response relationship between the HEI-2015 and OAB, as illustrated in Figure 2. The *P*_nonlinear value for HEI-2015 and OAB was determined to be 0.0257, indicating the selection of a non-linear relationship model. In the dose–response RCS plot, it was observed that the risk of OAB generally decreased with increasing HEI-2015 scores, displaying a likely “W” shape between them. The cutoff value for the body fat distribution parameters corresponded to the median values: 54.43.

### Subgroup analyses

The results of subgroup analyses across different groups are presented in Table 4. The findings indicated that BMI, gender, hypertension, smoking, and alcohol use may not have an impact on the relationship between HEI-2015 and OAB (*p* for the interaction >0.05). However, the association of HEI-2015 and OAB might be influenced by diabetes, wherein HEI-2015 exhibited a significant favorable association with individuals suffering from diabetes (OR = 0.973, 95%CI: 0.956, 0.991).

**Table 4:** Subgroup analyses between the association of HEI-2015 and OAB.

Character	OR 95% CI	*p* value	*p* for interaction
BMI groups			0.5
< 25	0.996 (0.983, 1.008)	0.491	
25–29.9	0.992 (0.979, 1.006)	0.256	
≥ 30	0.988 (0.975, 1.001)	0.060	
Gender			0.863
Male	0.994 (0.985, 1.003)	0.167	
Female	0.984 (0.970, 0.998)	0.027	
DM			0.006
No	0.995 (0.986, 1.004)	0.263	
Yes	0.973 (0.956, 0.991)	0.005	
Hypertension			0.057
No	0.996 (0.984, 1.008)	0.487	
Yes	0.985 (0.974, 0.996)	0.008	
CVD			0.103
No	0.992 (0.982, 1.001)	0.083	
Yes	0.977 (0.960, 0.994)	0.010	
Smoke			0.241
Never	0.994 (0.982, 1.006)	0.322	
Former	0.990 (0.975, 1.006)	0.214	
Current	0.975 (0.958, 0.994)	0.009	
Alcohol user			0.933
Never	0.993 (0.975, 1.011)	0.423	
Moderate	0.991 (0.980, 1.002)	0.121	
Heavy	0.988 (0.974, 1.003)	0.123	

OR, Odds ratio. A *p* for interaction > 0.05 indicated that the subgroup was meaningless.

## Discussion

Diet may be a risk factor or a protective factor of OAB. The Mediterranean diet has been reported to alleviate OAB symptoms (8). However, caffeinated beverages, spicy foods, and acidic foods may increase bladder sensitivity (9). In this topic, there is a lack of study on the general diet evaluation score and the association of OAB. To the best of our knowledge, this is the first cross-sectional study investigating the association between HEI-2015 and OAB in a large population. A higher continuous HEI-2015 value was independently linked to a lower OAB incidence (OR: 0.87; 95% CI: 0.78, 0.98; *p* = 0.02). Similarly, the highest quartile categorical HEI-2015 was significantly associated with lower OAB odds (OR: 0.72; 95% CI: 0.52, 0.99) compared to the lowest quartile. The downward trend in the RCS curve further supported the main outcome. These results suggest that adherence to the HEI-2015 may play a crucial role in the prevalence of OAB.

The current literature lacks comprehensive investigation into the mechanisms behind the association of diet quality and the development of OAB. We attempted to propose several potential theories. Firstly, previous studies have indicated heightened sensitivity of bladder sensory nerves or other neurological disorders in OAB (22). In our subgroup analysis, this study suggested that patients with diabetes mellitus might have OAB. Wang et al. reported that diabetic complications, including diabetic neuropathy and atherosclerosis, lead to systemic inflammation and urothelial nerve dysfunction (23). Consequently, we hypothesize that a low-quality diet (indicated by a low HEI-2015 score) may decrease the excitatory neurotransmission of the bladder, potentially leading to OAB. Secondly, obesity is a known primary cause of OAB. Individuals with a low-quality diet may be more prone to obesity. The accumulation of adipose tissue in the abdominal area, including the pelvic floor, increases intra-abdominal and intravesical pressure. Moreover, individuals with central obesity may simultaneously experience abdominal muscle sarcopenia (24). These factors can collectively decrease bladder capacity and voiding pressure, potentially leading to OAB. Thirdly, it has been reported that a Mediterranean diet, a low-salt diet, and higher consumption of bread and vegetables are beneficial for OAB symptoms (8, 25, 26). Considering this, we propose that a low HEI-2015 score (indicative of a high content of saturated fatty acids) might promote the production of pro-inflammatory cytokines, thus contributing to systemic inflammation—a potential mechanism of OAB (27, 28). Lastly, individuals with a low HEI-2015 score may be susceptible to atherosclerosis. Arterial atheromatous plaques in the bladder wall may cause detrusor ischemia, potentially leading to detrusor underactivity and ultimately OAB (29, 30). During the past 2 decades, there were emerging studies on dietary factor and OAB. The Leicestershire MRC Incontinence Study Group reported that most diet and factors were not associated with OAB while beer might have a protective role in men with OAB (31). Another study prospective suggested that causal associations with carbonated drinks was confirmed for OAB disorders in women (25). A recent animal study reported that a high salt diet impairs the bladder epithelial barrier to induce an overactive bladder (32). Compared with a previous single food factor, the HEI-2015 can give the healthcare providers or nutritionists a general and comprehensive evaluation of total food intake. While individual responses to diet can vary, making mindful dietary choices can play a crucial role in managing OAB symptoms. We may use the HEI-2015 to investigate the quality of total diet intake and OAB development in different populations in the future. In the clinical practice, if an OAB patient come with low HEI-2015 score, the physician or general practitioners can analyze the detailed HEI-2015 score, find the certain items which lead to a lower sore, then give the OAB patient a personalized suggestion and recommendation to improve the HEI-2015 score.

The management of OAB primarily focuses on alleviating symptoms. Recognizing the risk factors associated with the development of OAB is crucial. Healthcare professionals can utilize specific dietary indices to assess the quality of daily food intake (33). In addition to maintaining physical activity levels and fluid intake, dietary patterns reflect nutrient intake and may serve as a predictive factor for OAB. Recently, several studies have investigated the association of dietary/lifestyle factors and OAB. A study in turkey including 326 patients concluded that the Mediterranean diet was associated with lower OAB risk because that dietary pattern was poor in fat, thus reduce the risk of a factor of OAB, obesity (8). Another cohort study reported that high protein intake was associated with OAB symptoms. However, there was no significant relationship with carbohydrate and monounsaturated/polyunsaturated fat intake (34). A cross-sectional study using NHANES data emphasized the potential advantages of a high intake of active microbiota for preventing OAB (35). The antioxidant properties of vegetables/fruits may influence microbiota then influence OAB symptoms. Another NHANES research suggested that consuming a diet rich in flavonoid subclass anthocyanidin and flavone was associated with a reduced risk of OAB (36). There was also a strong positive correlation between food insecurity and the prevalence of OAB from a cross-sectional study including 29,129 participants (37). Other than dietary factors, lifestyle factors are also essential in OAB prevention. A recent study showed a significant negative correlation between LE8 scores and OAB prevalence, shedding light on the potential link between exercise/cardiovascular health and OAB (38).

The adoption of a concise form comprising 13 components of the HEI-2015 can enhance participants’ awareness of their diet quality. It is easy and convenient to determine outpatients’ diet by using the 13-item components HEI-2015 form. We believe that the recommendation of a dietary pattern with high HEI-2015 score should be the first line to the prevention and treatment of OAB. The HEI-2015 has been widely employed in the prevention and treatment of various diseases. This study contributes additional evidence regarding the link between dietary quality and OAB, which may assist community physicians in providing dietary guidance to patients with OAB.

We utilized the NHANES national multi-ethnic survey data and sample weighting to obtain representative results for the United States population. A key novelty of this study is the identification of a non-linear dose–response association between HEI-2015 and OAB. Despite its strengths, the study also has limitations. First, the calculation of OABSS value relied on a questionnaire interview within a real-time hospital setting, potentially introducing some degree of recall bias. Second, the absence of urodynamic results and laboratory tests for OAB patients in the NHANES data prevented the evaluation of the relationship between body fat distribution and the severity of OAB symptoms. Third, while our study included 29,206 participants, among whom 6,184 were diagnosed with OAB, the cross-sectional design makes it challenging to establish a causal relationship. In order to address these limitations, further researches involving more prospective cohorts across different racial groups and comprehensive meta-analyses investigating the relationship between HEI-2015 and OAB are urgently needed.

## Conclusion

In conclusion, our findings demonstrate a positive association between a higher HEI-2015 score and OAB, revealing a non-linear dose–response relationship. Consequently, we recommend adherence to United States dietary recommendations as a potential behavioral approach for OAB prevention. However, further large-scale long term prospective cohort studies across various regions are necessary to validate the outcomes of this investigation. Prospective studies on the HEI-2015 change and the afterwards influence on OAB are also needed.

## Data Availability

The original contributions presented in the study are included in the article/Supplementary material, further inquiries can be directed to the corresponding author.
